# Event-Related Alpha Suppression in Response to Facial Motion

**DOI:** 10.1371/journal.pone.0089382

**Published:** 2014-02-19

**Authors:** Christine Girges, Michael J. Wright, Janine V. Spencer, Justin M. D. O’Brien

**Affiliations:** Department of Psychology, Brunel University, London, United Kingdom; University of Tuebingen Medical School, Germany

## Abstract

While biological motion refers to both face and body movements, little is known about the visual perception of facial motion. We therefore examined alpha wave suppression as a reduction in power is thought to reflect visual activity, in addition to attentional reorienting and memory processes. Nineteen neurologically healthy adults were tested on their ability to discriminate between successive facial motion captures. These animations exhibited both rigid and non-rigid facial motion, as well as speech expressions. The structural and surface appearance of these facial animations did not differ, thus participants decisions were based solely on differences in facial movements. Upright, orientation-inverted and luminance-inverted facial stimuli were compared. At occipital and parieto-occipital regions, upright facial motion evoked a transient increase in alpha which was then followed by a significant reduction. This finding is discussed in terms of neural efficiency, gating mechanisms and neural synchronization. Moreover, there was no difference in the amount of alpha suppression evoked by each facial stimulus at occipital regions, suggesting early visual processing remains unaffected by manipulation paradigms. However, upright facial motion evoked greater suppression at parieto-occipital sites, and did so in the shortest latency. Increased activity within this region may reflect higher attentional reorienting to natural facial motion but also involvement of areas associated with the visual control of body effectors.

## Introduction

The visual system can reconstruct a perceptual scene from motion cues alone. For example, a human walker can be detected from just a dozen moving dots [1]. This perception of biological motion (BM) may underlie many aspects of social cognition [2]–[4]. Indeed, individuals can categorize genders, identify different people and recognize emotions from point-light walkers [5]–[8].

Neuroimaging studies suggest that BM data is processed within the superior temporal sulcus (STS) [9]–[12]. This substrate is a convergence point for dorsal and ventral pathways, and has multimodal associations with the amygdala, fusiform gyrus, MT+/V5 and cerebellum [10], [13]–[16]. Electrophysiological investigations support these data, in addition to providing a timeline of processing events [17]–[21]. Jokisch *et al.,* [22] compared event-related potentials (ERPs) evoked by upright, inverted and scrambled point-light walkers. The amplitudes of the early N170 component only differed between upright and inverted BM. This difference was not observed for the second negative peak (N300), which appeared to occur over the STS complex. The authors suggest that this late processing stage is specific to the visual analysis of BM. Krakowski *et al.,* [23] report similar findings. Compared to scrambled motion, viewing BM produced a positive shift of the ERP between 100 and 200 ms. This was followed by negativity from 200 to 350 ms over posterior middle temporal regions. Source analysis indicated that the first phase was generated by substrates involved in motion processing (MT+/V5), whilst neuronal populations within and around the STS evoked the negative-going ERP [23].

Whilst BM refers to both body and facial movement, there is little research concerning the latter. It is crucial however to extend our investigations to include this stimulus class, especially if we consider its prominent role in socio-emotional communication [2]. The present study thus examined facial motion perception via analysis of changes within the EEG alpha band (8–12 Hz). Originating from occipital regions, alpha waves are suppressed during active visual perception [24]–[25]. They appear to be synchronized with cyclic activity of the visual thalamic relay neurons, modulating signal transmission during early input stages [26]. Occipital alpha may also index memory processes, including those related to working memory loads and long-term stores [27]–[29]. Parieto-occipital alpha is also influenced by visual attention [30]–[33]. For example; alpha power is larger over visual cortices when attention is focused on the auditory part of an auditory-visual stimulus [34]. Participants also show an interhemispheric difference in alpha amplitudes during the Posner cueing paradigm [35]. The increase on the unattended side suggests alpha waves have a ‘gating mechanism’ [36]. Such function may inhibit incoming sensory information in terms of its behavioral relevance [37]–[38]. Others note that prestimulus alpha fluctuates with the excitability of the visual cortex and is predictive of an imminent perception [39]–[40].

Many studies have investigated static face perception by observing alpha oscillations [41]–[44]. Emotional faces increase alpha amplitudes at posterior occipital locations, whilst angry face stimulation specifically activates substrates over electrodes T5, P3 and O2 [45]. Frontal alpha activity is also associated with previously formed concepts concerning negative facial expressions, suggesting that the fronto-thalamic system is involved in the perception and evaluation of facial expressions [46], [47]. Regarding general face perception, Hsiao *et al.,* [48] found 4–25 Hz activity in the middle occipital and occipitotemporal areas when participants viewed upright faces. Inverted faces however produced the most alpha enhancement in the right occipitotemporal area, indicating additional attentional requirements and increased synchrony between neuronal populations. Sakihara *et al.,* [49] also found alpha, theta and beta suppression occurring over occipitotemporal areas during familiar, unfamiliar and own face perception. Such activity may illustrate the structural and semantic encoding of facial information [49], [50].

To our knowledge, no published EEG study has directly examined posterior alpha suppression in response to whole-face human motion. Participants therefore observed CGI averaged faces animated with human motion sequences. These exhibited both rigid (head rotations and translations) and non-rigid (facial expressions) motion, as well as speech expressions. Their task was to discriminate between successive motion captures during EEG recordings. Changes in alpha power were measured over parieto-occipital and occipital regions.

Further, the current study did not use inanimate (object) motion as a control. Such comparisons involve many unrestrained differences in low-level stimulus properties [51]. Instead, we used orientation-inverted and luminance-inverted facial stimuli as these manipulations are known to affect face recognition. Inversion paradigms impair static face perception by disrupting configural processing [52]. Moreover, the brain may treat orientation-inverted faces as objects, considering the involvement of the lateral occipital area here [53]. Luminance-inverted faces also affect processing regardless of preserving normal face structure and spatial frequencies [54], [55]. These negative images disrupt the N170 face-selective component and therefore early structural encoding [56], [57]. Together, these measures comprise an effective tool in evaluating facial motion perception [58], [59].

## Materials and Methods

### Ethics Statement

Ethical approval was obtained from Brunel University (Psychology Ethics Committee). Participants were given a description of the study and written informed consent was obtained.

### Participants

Nineteen individuals (9 male, 10 female, *mean age* = 28.53 years, *range* = 22–54 years) with normal or corrected-to-normal vision participated in this study. None of the sample had any history of neurological or psychological disorders.

### Stimuli

**Figure 1:**
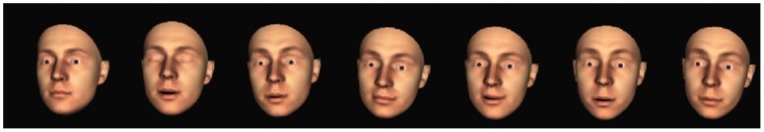
Snapshot series demonstrating part of a facial motion sequence.

The stimuli were taken from a video database developed by Hill and Johnston [59] using motion capture technology. Using markers placed on major facial landmarks, motion was captured from 12 actors reciting simple question and answer jokes. These jokes allowed natural facial expressions (non-rigid motion), speech and head movements (rigid motion) to be captured. The motion sequences were then applied to a three dimensional computer-generated averaged head (taken from 100 men and 100 women) and outputted as 640×480 pixels, 25 frames-per-second movies (Figure 1, but see video S1 for a dynamic example). By using an average face on all sequences, facial motion could be measured independently from structural and surface-based facial cues. The appearances of all motion capture faces were therefore identical, and only differed in the way they moved. An orientation-inverted and luminance-inverted version of each stimulus was generated in Matlab (MATLAB 7.10.0. Natick, Massachusetts: The MathWorks Inc., 2010) by manipulation and re-encoding of the original stimulus video file.

### Procedure

Observers viewed the dynamic stimuli on a computer screen. Viewing distance was 80 cm, at which the distance of the 38 cm×30 cm display subtended an angle of approximately 28°×22°. The experiment consisted of 3 blocks, each with 50 trials; upright facial motion, orientation-inverted facial motion and luminance-inverted facial motion. Blocks were repeated three times in a counterbalanced order to avoid practice effects, fatigue or decreasing vigilance influencing the EEG waveform.

Participants completed a *sequence discrimination task* during the EEG recordings. This tested their ability to differentiate between facial motion sequences that were presented in a continuous series. All animations were presented for 3000 ms. A single animation was presented, and after an interstimulus interval (ISI) of 1000 ms, another animation appeared. During a second ISI, participants were required (according to pre-task instructions) to respond via the keypad, whether the two animations were the same (press 1) or different (press 2) from each other. This process continued throughout the testing period, such that they always judged whether the current animation was the same or different from the previous animation. The same format was used for all three conditions (upright, orientation-inverted and luminance-inverted).

### ERP Recording and Analysis

EEGs were recorded with an average common reference from 64 Ag-AgCl electrodes. These were placed according to the International 10/20 system. A horizontal electrooculogram (EOG) was recorded from electrodes placed on the outer canthi of both eyes. Vertical EOG electrodes were placed above and below the middle of the left eye. Impedances did not exceed 10 KΩ. The EEG was amplified at a gain of 1000 and bandpass filtered at 0.1–100 Hz. It was digitized at 1000 Hz via a Synamps2 amplifier and Scan 4.4 acquisition and analysis software (Compumedics Neuroscan Ltd.).

Offline, a DC offset correction was applied to the raw waveform, and the time series was bandpass filtered at 0.1–128 Hz (24 dB/octave). A visual scan was conducted to mark ‘bad’ blocks and eye blink artifacts were removed by a principle components procedure. Using the cleaned EEG, an event file was created and used to epoch the data for each condition from −100 to 923 ms (0 ms = stimulus onset). Sweeps were baseline corrected (entire sweep) and amplitudes greater than ±75 µV were rejected. An event-related band power analysis detected event-related frequencies within the alpha band. The data was band-pass filtered with a centre frequency of 10 Hz, and a half bandwidth of 2 Hz (12 dB/octave) within a moving 100 ms window. The baseline, mid-point maximum and late-minimum amplitudes (and the latency in which they occurred), were detected and analyzed.

### Statistical Analysis

A repeated-measures ANOVA was used to test for differences between the alpha amplitudes elicited by each facial motion. *Time sample* (baseline, mid-point, late-minimum), *sequence type* (same, different) *face type* (upright, orientation-inverted, luminance-inverted), *hemisphere* (left, right) and *electrode site* were the within-participant factors. A Bonferroni correction was applied to post-hoc contrasts. A repeated-measures ANOVA (*face type*×*hemisphere*×*electrode site*) was used to analyze differences in the latencies of alpha amplitudes produced by each face type. As *sequence type* did not yield any significant main effects, data was collapsed across these levels.

## Results

The strongest alpha power was observed at parieto-occipital (PO7, PO5, PO3, PO8, PO6, PO4) and occipital (CB1, O1, O2, CB2) scalp locations. Amplitudes were observed at three time samples (*baseline* −100–0 ms, *mid-point* 300–500 ms, and *late-minimum* 600–823 ms). Observing data at three time-samples allowed a more detailed analysis to be made with regards to patterns of alpha activity post motion onset.

### Grand Average Data

Data from one participant were excluded from statistical analysis due to technical faults with the EEG recording system. At parieto-occipital (PO) and occipital (O) sites, upright facial motion increased alpha power before suppressing it. This pattern of alpha activity did not occur for other stimuli (Table 1).

**Table 1:** Grand averaged amplitude and latency data for facial motion at PO and O sites.

Site	Face type	Baseline*	Mid-point	Lateminimum
*PO*	*Upright*	4.77 µV at0 ms	4.94 µV at477 ms	2.71 µV at733 ms
	*Orientation-Inverted*	4.52 µV at0 ms	3.82 µV at466 ms	2.80 µV at755 ms
	*Luminance-inverted*	4.56 µV at0 ms	4.22 µV at453 ms	2.97 µV at734 ms
*O*	*Upright*	5.24 µV at0 ms	5.38 µV at443 ms	3.48 µV at731 ms
	*Orientation-Inverted*	5.07 µV at0 ms	4.27 µV at467 ms	3.05 µV at754 ms
	*Luminance-inverted*	5.17 µV at0 ms	4.63 µV at460 ms	3.40 µV at747 ms

*Baseline amplitudes are considered the initial values of alpha.

### Amplitude Data

Facial motion (regardless of type) suppressed alpha power, as indicated by significant differences between the *time-sample* amplitudes (*O sites: F*_ (2, 16)_ = 32.45, *p*<0.01 and *PO sites: F*_ (2, 16)_ = 52.95, *p*<0.01). For PO data, simple contrasts indicated a significant difference between the late-minimum interval and baseline for all facial motion (*Upright F*_ (1, 17)_ = 41.68, *p*<0.005; *Luminance-inverted F*_ (1, 17)_ = 90.71, *p*<0.005; *Orientation-inverted F*_ (1, 17)_ = 39.43, *p*<0.005). The difference between the recovery interval and baseline was significant for orientation-inverted faces only (*F*_(1, 17)_ = 9.88, *p*<0.05). At PO and O electrodes, there was a significant main effect of *face type* on overall alpha power across the three time-samples (*F*_(2, 16)_ = 3.97, *p*<0.05 and *F*_ (2, 16)_ = 4.67, *p*<0.05, respectively).

The amount of alpha suppression evoked by each facial motion only differed at PO sites, as revealed by a significant *time-sample*×*face type* interaction (*F*_(4, 14)_ = 6.39, *p*<0.01). Simple contrasts showed that this interaction was driven by a significant difference between upright and orientation-inverted faces in the mid-point time interval only (*F*_(1, 17)_ = 11.64, *p*<0.05). See Table 2 for a summary of significant main effects and interactions.

**Table 2:** Significant main effects and interactions at PO and O electrodes.

Electrodes	Within-participant variables	*F*	*df*	*P*
**PO**	*Time-sample* *	52.95	2, 16	0.001
	*Face type* *	3.97	2, 16	0.040
	*Time-sample*×*face type* *	6.39	4, 14	0.001
	*Electrode site*	33.06	2, 34	0.001
	*Time-sample*×*hemisphere*	4.81	2, 34	0.014
	*Sequence*×*face type*×*electrode* *	4.89	4, 14	0.011
**O**	*Time-sample* *	32.45	2, 16	0.001
	*Face type* *	4.67	2, 16	0.025

*Results taken from multivariate tests (Pillai’s Trace) due to a significant Mauchly’s test indicating that sphericity cannot be assumed.

### Latency Data

Differences in the latency of the peak alpha amplitudes were observed amongst the facial motion types (Table 3). *Face type* had a significant effect on the latency of the late-minimum amplitudes at PO sites (*F*_(2, 34)_ = 3.44, *p*<0.05). Simple contrasts indicated that this was driven by a significant difference between upright and orientation-inverted facial motion (*F*_(1, 17)_ = 6.27, *p*<0.05). Compared with other types, upright motion suppressed alpha at earlier latencies (733 ms vs. 755 ms for orientation-inverted stimuli). At O sites, the latencies of the mid-point amplitudes were significantly affected by *face type* (*F*_(2, 34)_ = 4.57, *p*<0.05). Simple contrasts revealed a significant difference between the mid-point latencies for upright (443 ms) and orientation-inverted (467 ms) facial motion (*F*_(1, 17)_ = 7.20, *p*<0.05).

**Table 3:** Latency of mid-point peak and minimum amplitudes at PO and O electrodes.

*Latency of mid-point peak amplitudes*				
Site	Within-participant variables	*F*	*df*	*P*
PO	Hemisphere×electrode	4.04	1.42, 24.07*	0.043
O	Face type	4.57	2, 34	0.018
	*Upright vs. orientation-* *inverted*	7.20	1, 17	0.016
*Latency of late-minimum amplitudes*				
PO	Face type	3.44	2, 34	0.044
	*Upright vs. orientation-* *inverted*	6.27	1, 17	0.023
	Electrode	10.15	2, 34	0.001
O	Hemisphere	8.58	1, 17	0.009

*Mauchly’s test indicated that the assumption of sphericity had been violated. Degrees of freedom were therefore corrected using Greenhouse-Geisser estimates of sphericity.

## Discussion

### Transient Alpha Increase

Unexpectedly, upright facial motion initially evoked an increase in alpha over parieto-occipital and occipital regions. The neural efficiency argument [60] provides one interpretation for this. Reflecting on our expertise, less information processing is required for upright face perception [61]. This would certainly explain why the control faces evoked alpha suppression almost instantly; unfamiliar stimuli would require increased attentional effort and involvement of high-level cognitive resources [54], [62]. Yet this argument does not explain why upright facial motion subsequently suppressed alpha after this time point. Alternatively, the initial high alpha amplitude could reflect a ‘gating’ mechanism used to filter out irrelevant visual inputs [37], [38]. In this case, form cues provided no additional information and were thus ignored. The subsequent suppression would therefore correlate with attention to motion cues when engaging in facial motion tasks. It is important to note however that this transient increase in alpha following video onset could be due to the motion-onset ERP. As our data analysis was conducted using induced event-related bandpower measures, we cannot fully address this point. Future studies could potentially utilize evoked synchronization measures in order to observe a more distinct emergence of face-selective ERP components.

### Posterior Activation in Facial Motion Perception

With reference to the amount of suppression evoked by each facial motion, no difference emerged at occipital locations. This suggests that early visual processing occurs irrespective of orientation or luminance-reversal [47], [63]. This finding is in contrast to studies of static face perception. For example, Itier and Taylor [56] report that inverted and negative faces affected early encoding, as demonstrated by a reduced N170 response. Further, in the context of encoding and retrieval mechanisms, occipital alpha is suppressed when participants perceive famous (and thus familiar) faces compared to non-famous faces [64]. The authors suggest face perception evokes interplay between semantic knowledge and episodic memory formation. In the case of biologically unfamiliar faces (e.g., orientation-inverted or luminance-inverted stimuli), we may expect less occipital alpha activity to occur. Yet, this effect was not found here. It is possible that early encoding processes remain unaffected by such visual manipulations, perhaps due to the detailed three-dimensional representation facial motion provides [65]. We are disinclined to accept this view however as many studies do report a disruption in perceiving inverted point-light figures [22], [66], [67]. To our knowledge, only one study has found a comparable response to upright and inverted walkers over the left occipital cortex [68].

By contrast, upright facial motion reduced alpha more than control stimuli at parieto-occipital regions. A study comparing the ERP response to upright and scrambled point-light walkers also reports differences emerging over this region [23]. In addition, stronger alpha suppression following BM perception has been noted over the parieto-occipital cortex [69]. This enhanced activity may reflect a number of significant underlying processes. First, the medial portion of the parieto-occipital cortex has been associated with attentional reorienting during cognitive-motor tasks [70]. Others extend this finding to the dorsal aspect of the parieto-occipital sulcus [71]. An increase in parieto-occipital activity thus suggests higher attentional effort allocated to perceiving upright facial motion. Second, the parieto-occipital cortex contains functional areas associated with the visual control of body effectors [72]. Regions of the superior and medial portions play a critical role in proximal and distal aspects of reaching/grasping movements, pointing gestures, head movements and eye-gaze shifts [73]–[75]. Motion selectivity has also been observed within this region [76], indicating dorsal visual stream involvement [77]. Perhaps observing upright facial motion, which included head and eye translations, activated a portion of these substrates. Further, it is possible that parieto-occipital electrodes are indirectly recording activity occurring within the posterior superior temporal sulcus (pSTS). One study which found face-selective ERPs occurring over the parieto-occipital cortex to facial motion supports this idea [78]. There is also evidence that the STS may actually extend into the parieto-occipital and occipital regions [79].

The larger amount of suppression evoked by upright facial motion also occurred within the shortest latency at parieto-occipital sites. Such early processing could reflect a pop-out effect caused by familiar orientations [22]. If this was the case though, luminance-inverted faces would have also been processed just as quickly. Instead, automated feed forward systems may in part be responsible for the efficient processing of upright motion [9], [80]–[82]. Yet, top-down computations should not be completely disregarded [83]. For example, body motion perception utilizes a feedforward and feedback functional loop between the right pSTS and left lateral cerebellum [15].

### Implications of the Current Data

The results may have been influenced by sensorimotor alpha (mu rhythms) recorded over central electrodes. Mu rhythms index action planning and preparation within the somatosensory cortex [84], [85]. They are suppressed and their power attenuated when one performs an action [86] but also during the observation of biological movements [87]–[89]. In the current study, participants responded via a button press after observing facial motion sequences. Such experimental paradigm perhaps activated anterior systems. It should be noted however that central electrodes were analyzed, and no significant effects found.

In addition, we did not use inanimate or scrambled motion as a control. Thus, it remains unknown whether differential activations would have occurred for any stimuli presented in unfamiliar contexts. However, the manipulations we used are known to disrupt configural and holistic processing, meaning that control stimuli may be processed in a manner similar to objects [53]. We are disinclined then to suggest a role of familiarity, but instead that the parieto-occipital region shows selectivity to upright facial motion.

## Supporting Information

Video S1 **Example of the facial motion animations used in this study.** Please see Hill and Johnston [59] for further examples and a full description of how the stimuli were made.
(AVI)
